# A calibration curve for immobilized dihydrofolate reductase activity assay

**DOI:** 10.1016/j.dib.2015.04.005

**Published:** 2015-04-20

**Authors:** Priyanka Singh, Holly Morris, Alexei V. Tivanski, Amnon Kohen

**Affiliations:** The Department of Chemistry, The University of Iowa, Iowa City, IA 52242, USA

**Keywords:** Dihydrofolate reductase, Activity assay

## Abstract

An assay was developed for measuring the active-site concentration, activity, and thereby the catalytic turnover rate (*k*_cat_) of an immobilized dihydrofolate reductase model system (Singh et al., (2015), *Anal. Biochem*). This data article contains a calibration plot for the developed assay. In the calibration plot rate is plotted as a function of DHFR concentration and shows linear relationship. The concentration of immobilized enzyme was varied by using 5 different size mica chips. The dsDNA concentration was the same for all chips, assuming that the surface area of the mica chip dictates the resulting amount of bound enzyme (i.e. larger sized chip would have more bound DHFR). The activity and concentration of each chip was measured.

## Specifications table

**Table:** 

Subject area	*Chemistry*
More specific subject area	*Biochemistry*
Type of data	*Figure*
How data was acquired	*HPLC* (*Agilient 1100 Series*), *Packard Tricarb Tr2900 liquid scintillation counter* (*LSC*)
Data format	*Analyzed data*
Experimental factors	*None applied*
Experimental features	*Reaction product was separated using reverse phase HPLC and radioactivity was counted on LSC*
Data source location	*Iowa City, Iowa, USA*
Data accessibility	*The data are with this article*

## Value of the data

• Novel technique to measure the concentration and activity of enzyme immobilized on a solid surface at sub-monolayer coverage [1]
• The approach has great potential of assisting in the analytical determination of active site concentration of a wide variety of immobilized enzymes on solid surfaces or nanoparticles
• The calibration plot suggests linear relationship between rate as a function of enzyme concentration

## 1 Data, experimental design, materials and methods

### 1.1 Immobilization procedure

*E*scherichia *coli* DHFR was expressed, purified, and stored as discussed elsewhere [2,3]. The labeled (thiol/amino terminated) dsDNA linkers were generated by PCR using *Taq* DNA polymerase and a pEt22b-DHFR vector as a template [3]. The primers used were 5′ thiol_TTA GCG GTA GAT CGC GTT ATC GGC ATG and 5′ amino _ TTC CCA GGT ATG GCG GCC CAT AAT CAC. DNA linkers (123 base pair; bp) were generated by annealing complementary oligonucleotides. The PCR products were purified using the QIAquick PCR purification kit from Qiagen. The labeled dsDNA linkers were attached to pre-activated mica using ethanolamine and 1,4-phenylene diisocyanate through the 5′ amino terminated end as described elsewhere [4–10]. Since the concentration of dsDNA linker used in the procedure was relatively small, complete surface coverage does not occur and ethanolamine was used to block any remaining reactive sites on the surface. After activation of the dsDNA containing thiol group with tris-(2-carboxyethyl)phosphine hydrochloride (TCEP) [11], the plate was finally submerged in activated DHFR in MTEN buffer (pH 7.5), ultimately resulting in disulfide bond formation between the thiol on DNA and a surface cysteine residue on the enzyme.

### 1.2 Atomic force microscopy imaging

A molecular force probe 3D AFM (Asylum Research, Santa Barbara, CA) was used to collect images. A silicon nitride Veeco probe (Model SNL-10) with nominal spring constant of 0.6 N/m was used for AC imaging of the sample. The mica substrate was fixed with epoxy to a liquid cell and the images were collected in MTEN buffer at room temperature.

### 1.3 Calibration plot

**Fig. 1:**
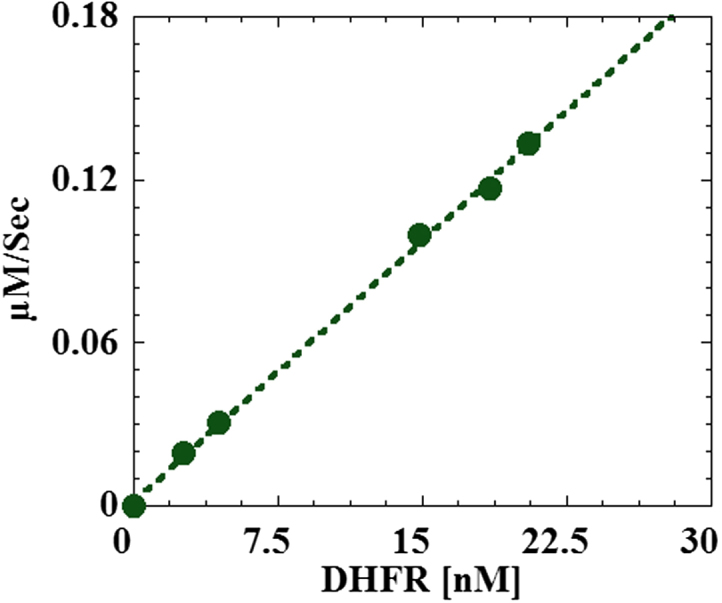
Rate in µM/S is plotted as a function of DHFR concentration. The plot shows linear relationship between DHFR concentration and rate.

For this experiment we varied the concentration of immobilized enzyme by using 5 different size mica chips. We kept the dsDNA concentration the same for all chips, assuming that the surface area of the mica chip dictates the resulting amount of bound enzyme (i.e. larger sized chip would have more bound DHFR). The activity and concentration of each chip was measured [12,13]. The plot of rate (µM/s) versus DHFR concentration (nM) can be seen in Fig. 1. The results show that rate as a function of concentration is in fact linear.

## Conflict of interest

The authors declare that there is no conflict of interest on any work published in this paper.
